# Shared Pathophysiology of Inflammatory Bowel Disease and Psoriasis: Unraveling the Connection

**DOI:** 10.7759/cureus.68569

**Published:** 2024-09-03

**Authors:** Walter Jauregui, Yozahandy A Abarca, Yasmin Ahmadi, Vaishnavi B Menon, Daniela A Zumárraga, Maria Camila Rojas Gomez, Aleeza Basri, Rohitha S Madala, Peter Girgis, Zahra Nazir

**Affiliations:** 1 General Medicine, Universidad Nacional Autónoma de Honduras, Tegucigalpa, HND; 2 Internal Medicine, Escuela de Medicina y Ciencias de la Salud, Tecnológico de Monterrey, Mexico City, MEX; 3 School of Medicine, Royal College of Surgeons in Ireland - Medical University of Bahrain, Muharraq, BHR; 4 Internal Medicine, Sri Ramachandra Institute of Higher Education and Research, Chennai, IND; 5 General Medicine, Universidad San Francisco de Quito, Quito, ECU; 6 School of Medicine, Universidad del Rosario, Bogotá, COL; 7 Internal Medicine, Liaquat University of Medical and Health Sciences, Hyderabad, PAK; 8 Dermatology, Dr. PSIMS and RF, Vijayawada, IND; 9 Internal Medicine, Ross University School of Medicine, Bridgetown, BRB; 10 Internal Medicine, Combined Military Hospital, Quetta, PAK

**Keywords:** infliximab, psoriasis, gut microbiome, crohn’s disease (cd), ulcerative colıtıs

## Abstract

Psoriasis (PS) and inflammatory bowel disease (IBD) are immune-mediated chronic conditions that share pathophysiological processes, including immune system dysfunction, microbiome dysbiosis, and inflammatory pathways. These pathways result in increased turnover of epithelial cells and compromised barrier function. The assessment of the literature suggests that immunopathogenic mechanisms, such as tumor necrosis factor (TNF)-α signaling and IL-23/IL-17 axis dysregulation, are shared by PS and IBD. Clinical characteristics and diagnostic approaches overlap significantly, and advances in biomarker identification benefit both conditions. Current treatments, namely biologics that target TNF-α, IL-17, and IL-23, show promising results in decreasing inflammation and controlling symptoms. Precision medicine approaches are prioritized in prospective therapeutic procedures to tailor pharmaceuticals based on specific biomarkers, perhaps improving outcomes and minimizing side effects. This study thoroughly examines and evaluates the body of research on PS and IBD. Several papers were examined to compile data on clinical features, diagnosis, therapies, pathophysiology, epidemiology, and potential future therapeutic developments. The selection of articles was based on three methodological qualities: relevance and addition to the knowledge of IBD and PS. The retrieved data were combined to provide a coherent summary of the state of the knowledge and to spot new trends. The overview of the latest studies demonstrates that both PS and IBD share pathophysiological foundations and therapeutic approaches. With a spotlight on particular biomarkers, advances in precision medicine provide a promising path toward enhancing therapeutic effectiveness and minimizing side effects.

## Introduction and background

Psoriasis and inflammatory bowel disease (IBD) are chronic, immune-mediated conditions that significantly impact the quality of life for millions worldwide. It is estimated that around 60 million individuals worldwide have psoriasis [1]. In the United States alone, about three million people live with IBD [2]. Despite affecting different primary organ systems, emerging evidence suggests a substantial overlap in these two diseases' pathophysiological mechanisms and clinical manifestations [1,3-5].

Psoriasis is a long-lasting inflammatory skin condition characterized by a significant genetic predisposition and autoimmune pathogenic features [6]. In individuals with a genetic predisposition, multiple triggers such as stress, seasonal variations, infections, and sun exposure can provoke the onset of the disease [7]. The dermatologic expressions of psoriasis are diverse; there are several clinical subtypes of psoriasis, with chronic plaque psoriasis (psoriasis vulgaris) being the most prevalent form of the condition, representing approximately 90% of all cases [8]. Chronic plaque psoriasis is characterized by pruritic plaques veiled with silvery scales, making it easily recognizable [9]. Less frequently observed types of psoriasis encompass guttate, erythrodermic, and pustular psoriasis. Guttate psoriasis, which comprises 2% of all cases, manifests as numerous small scaly papules arranged in a central pattern [10].

IBD is caused by chronic inflammatory disorder; it comprises Crohn's disease (CD) and ulcerative colitis (UC). It features a progressive and unpredictable disease course marked by symptoms such as abdominal pain, diarrhea, bloody stools, and weight loss [11,12]. CD and UC can vary widely in their progression, ranging from mild cases with occasional symptoms to severe conditions requiring hospitalization and surgery and may lead to impairment [13,14]. UC is characterized by diffuse, continuous colon inflammation extending from the rectum proximally. On the other hand, CD can occur anywhere from mouth to anus, with the terminal ileum and cecum being the most commonly involved areas. While the precise cause of IBD remains uncertain, it is believed to arise in individuals with a genetic predisposition, potentially triggered by undefined environmental factors, changes in the gut microbiome, and immune system deregulations [15,16].

IBD and psoriasis exhibit a complex interplay of genetic predispositions, immune system dysregulation, environmental triggers, and microbiome alterations [4]. Genome-wide association studies (GWAS) have identified numerous susceptibility loci shared between IBD and psoriasis, highlighting a genetic overlap that underscores their common pathogenic pathways [4,17,18]. The Th17 cell-mediated immune response and the IL-23/Th17 axis play pivotal roles in the inflammatory processes of both conditions [19]. Additionally, dysbiosis in the gut microbiome has been implicated in the pathogenesis of IBD and psoriasis, further emphasizing the interconnected nature of these diseases [20-23].

Understanding the shared mechanisms between IBD and psoriasis is crucial for developing integrated management strategies to improve patient outcomes. This review explores the epidemiological trends, genetic factors, environmental influences, and treatment modalities associated with IBD and psoriasis. By unraveling the commonalities in their pathophysiology, we seek to provide insights that could pave the way for novel therapeutic approaches and enhance the improved care for patients suffering from these debilitating conditions.

## Review

Psoriasis

Psoriasis is a chronic autoimmune skin disease characterized by multiple systemic inflammatory features [24-26]. Psoriasis affects up to 3% of the global population, impacting life for those affected [27]. In Western countries, the prevalence ranges from 2-4%, with regional incidence rates varying from 0.09% to 11.43% [28]. Factors such as age, gender, geography, and ethnicity influence the prevalence of psoriasis, likely due to genetic and environmental factors. Higher prevalence rates have been reported among Caucasians and populations living at higher altitudes [29]. Approximately one-third of patients with psoriasis have first-degree relatives diagnosed with the same condition, indicating a significant genetic predisposition. The age of onset exhibits a bimodal distribution, with peaks occurring in males at 30-39 and 60-69 years old and approximately 10 years earlier in females [30].

**Figure 1:**
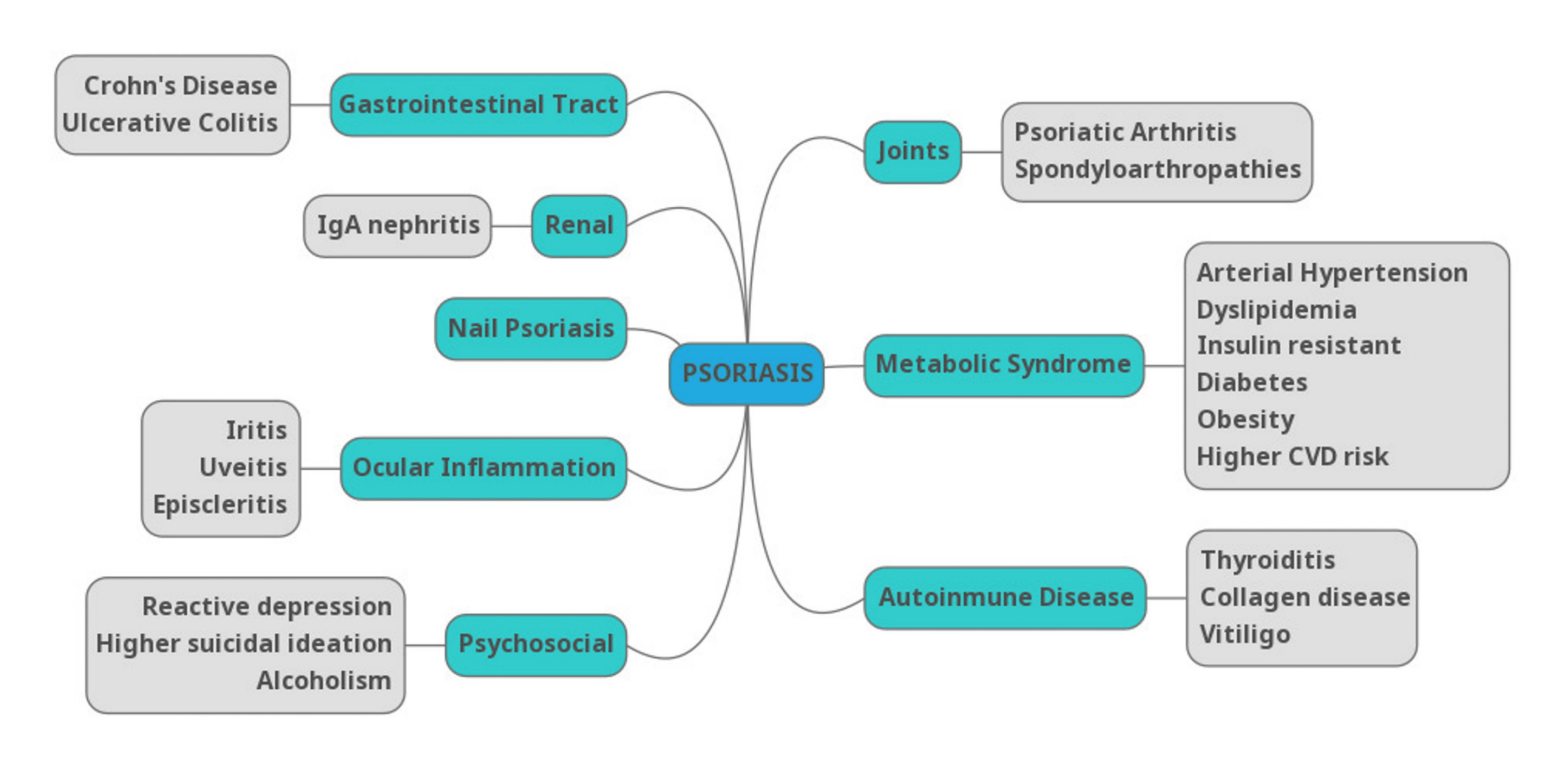
Psoriasis-associated comorbidities Reference: [24] CVD: cardiovascular disease

Psoriasis most commonly presents as chronic, symmetrical, erythematous, scaling, papules, and plaques, but it can present with many other clinical cutaneous manifestations [25]. Its onset and exacerbation are associated with many stressful physiological, psychological, and environmental factors [24]. Psoriatic disease is associated with several comorbidities (Figure 1), further complicating its clinical management.

Classification of psoriasis

Psoriasis can be classified into two primary types: pustular and non-pustular, each encompassing several subtypes, summarized in Table 1.

**Table 1:** Classification of psoriasis

Type	Subtype	Characteristics	Reference
Non-pustular psoriasis	Psoriasis vulgaris	It is characterized by erythematous, itchy, and sharply defined plaques that are covered in silvery scales, most commonly present on the scalp, extensor surfaces of the limbs, and the trunk.	[6]
	Guttate psoriasis	It is characterized by small drop-like scaly plaques having a diameter of 2 to 10 mm, usually 1-3 weeks after acute infection such as streptococcal tonsillitis.	[31]
	Erythrodermic psoriasis	It is a severe illness where more than 90% of the body's surface is swollen and erythematous, typical papules and plaques lose their characteristic features and require immediate medical attention.	[6,32]
	Palmoplantar psoriasis	It affects palms of hand and soles of feet symmetrically with more frequently involving thenar regions than hypothenar regions. Erythema is not always present, often if present appears as pinkish-yellow lesions.	[32]
	Psoriatic arthritis	It affects different tissues and clinical domains such as arthritis, spondylitis, enthesitis, and dactylitis). Early diagnosis and treatment can prevent joint destruction and disability.	[33]
	Inverse psoriasis	It’s characterized by slightly erosive erythematous plaques and patches affecting intertriginous areas. It is also called Flexural psoriasis.	[6]
Pustular Psoriasis	Pustular psoriasis	It is characterized by infiltration of neutrophil granulocytes in epidermis causing multiple sterile pustules which can present as localized and generalized conditions. Localized pustular psoriasis: palmoplantar pustular psoriasis (Barber type) and Acrodermatitis continua of Hallopeau. Generalized pustular psoriasis: von Zumbusch type.	[6,34]
	Impetigo herpetiformis	It appears as an erythematous lesion covered with pustules radiating from flexural regions with a tendency to form clusters. It might become more vegetative at skin folds.	[32]

Pathophysiology of psoriasis

Psoriasis is characterized by a chronic, relapsing-remitting course [35]. It results from immune system dysregulation triggered by endogenous danger signals and cytokines, leading to an autoimmune reaction in the epidermal layer of the skin [10]. Histologically, psoriatic plaques display acanthosis (epidermal hyperplasia) and inflammatory infiltrates composed of dermal dendritic cells, macrophages, neutrophils, and neovascularization [6].

The pathogenesis of psoriasis involves genetic predisposition, immune system dysregulation, and environmental triggers. Antigen-presenting cells, particularly dendritic cells, are critical in the initial stages of the disease [36]. These cells produce TNF-α and IL-23, promoting T cell differentiation toward Th17 cells, which secrete key psoriatic cytokines such as IL-17, IFN-γ, and IL-22. These cytokines contribute to skin inflammation, keratinocyte activation, and hyperproliferation, leading to psoriatic plaques [37].

**Figure 2:**
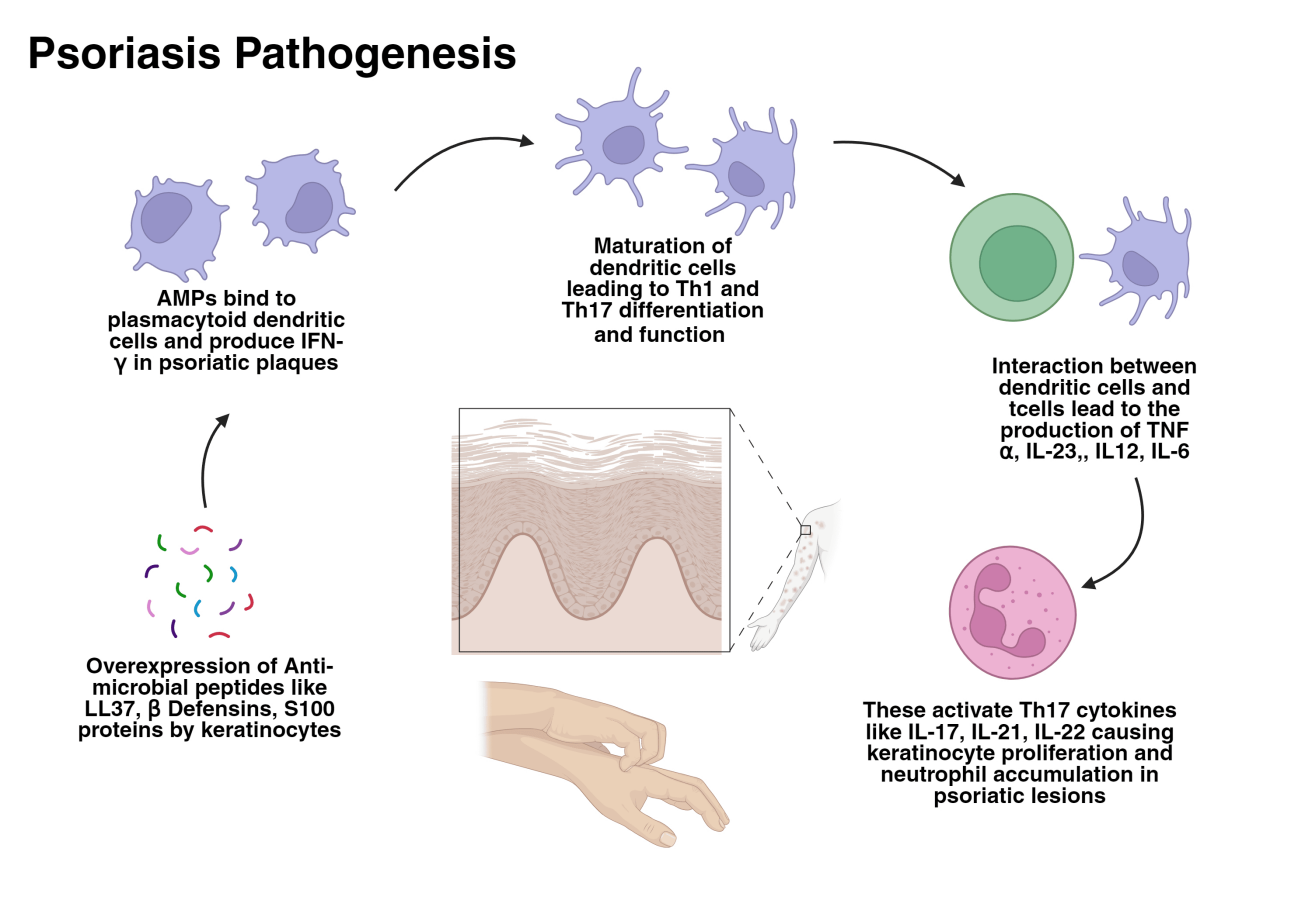
Inflammatory cases leading to psoriasis Reference: [36] AMPs: antimicrobial peptides

A key mechanism involves keratinocytes' overexpression of antimicrobial peptides (AMPs) in response to injury [6]. Among these, LL37, β-defensins, and S100 proteins are notable. LL37 binds to damaged keratinocytes, activating plasmacytoid dendritic cells to produce IFN-α in psoriatic plaques [36]. This leads to the maturation of myeloid dendritic cells implicated in Th1 and Th17 differentiation and function. When dendritic cells interact with T cells, resulting in the production of TNF-α, IL-23, IL-12, and IL-6, activating Th17 cytokines like IL-17, IL-21, and IL-22, causing keratinocyte proliferation and neutrophil accumulation in psoriatic lesions [6,36]. Figure 2 illustrates the pathogenesis of psoriasis.

The IL-17 cytokine family, especially IL-17A and IL-17F, plays a significant role in plaque-type psoriasis, with IL-17A exerting a more potent effect. In contrast, pustular psoriasis is more prominent in the innate immune system. In conclusion, the TNFα-IL23-Th17 axis is central to plaque psoriasis pathogenesis [38].

Genetic factors in psoriasis

Genetic studies have identified several susceptibility loci for psoriasis, with HLA-Cw6 being the most strongly associated. This genetic predisposition impacts immune system functioning, particularly the IL-23/Th17 axis [39]. Linkage studies have identified nine chromosomal segments (PSORS1-9) associated with psoriasis, with PSORS1 on chromosome 6p21.3 being the most significant, accounting for 35-50% of heritability. This region includes the major susceptibility allele HLA-Cw6, mainly linked to early-onset psoriasis. GWAS have identified over 40 regions associated with psoriasis, including essential genes such as IL12B, IL23R, and IL23A, underscoring the critical role of the IL-23/Th17 axis [40].

Microbiome in psoriasis

In healthy individuals, the gut microbiota offers several health benefits to the host, including protection against pathogens, support for nutrition and metabolism, and enhancement of the immune system. Most healthy individuals have a core microbiome set that is consistent regardless of diet, ethnicity, or culture [41]. Intestinal microbiota composition and functions are influenced by various factors, including the mode of delivery at birth, dietary habits, and antibiotic use, all of which play a crucial role in bacterial diversity [23]. An imbalance, known as dysbiosis, can trigger proinflammatory effects in the gut, skin, and joints [42]. These factors collectively contribute to the robustness or vulnerability of the gut microbiome. Over 160 species are identified, with Firmicutes and Bacteroidetes dominating and comprising about 90% of the stable gut microbiome [43]. The gut microbiota defends the host against bacterial infections by competing with pathogens for nutrients and producing bacteriocins, hydrogen peroxide, and short-chain fatty acids (SCFAs) such as butyrate, acetate, and propionate [44]. SCFAs produced by bacteria from these two phyla during anaerobic fermentation of dietary fiber are believed to have anti-inflammatory properties. Studies suggest that SCFAs can induce regulatory T cells in the colon, maintain homeostasis, and modulate the function of intestinal macrophages [45,46].

The development of psoriatic disease is significantly influenced by genetic and other factors that disrupt the gut or skin microbiome. These disruptions can lead to significant alterations in microbial composition, which may trigger disease onset. Both intestinal and skin microbial populations play a role in the pathogenesis of psoriatic disease [42]. In the gut microbiome of individuals with psoriasis, there is an imbalance in the ratio of Firmicutes to Bacteroidetes compared to healthy controls [47]. In the cutaneous microbiome among individuals with psoriasis compared to healthy individuals, there has been an observed upward trend in the prevalence of Streptococcus and a decline in levels of Propionibacterium [48]. These changes are thought to disrupt the skin barrier and promote local inflammation. 

Changes in diet can influence skin health by modifying the gut microbiome, suggesting an interconnection between gut and skin conditions, impacting them either positively or negatively [49]. Dietary interventions offer a therapeutic avenue for managing psoriatic symptoms. Chronic alcohol consumption disrupts gut microbiota balance, reducing Bacteroides while increasing Proteobacteria, Fusobacteria, and potentially pathogenic bacteria such as Prevotellaceae, Enterobacteriaceae, Veillonellaceae, and Streptococcaceae [50]. These alterations can exacerbate psoriatic symptoms and overall disease severity. Tobacco smoke also alters the gastrointestinal microbiome, which leads to decreased diversity and an increased presence of specific bacterial genera like Bacteroides, Prevotella, Enterobacteria, and Clostridium [42]. This further underscores the importance of lifestyle factors in the management of psoriasis.

Dysbiosis produces an abnormal immune response in psoriasis, with changes in the microbiome correlated with irregular inflammation-related markers in psoriasis patients. Specifically, the IL-2 receptor is positively associated with Phascolarctobacterium and negatively associated with Dialister [23]. These bacteria's relative abundances could be used as predictors of psoriasis activity. Additionally, complement three was negatively correlated with Escherichia levels, typically higher in psoriasis patients [23,42,51]. Butyrate plays a role in regulating inflammatory factors such as TNF-α, IL-10, and IL-1β. Psoriasis patients often show lower levels of beneficial butyrate-producing bacteria like *Faecalibacterium spp. *and higher levels of harmful bacteria like *Ruminococcus gnavus* [17,42,51].

Inflammatory bowel disease

CD

CD can manifest anywhere in the gastrointestinal tract, anywhere from mouth to anus, unlike UC, which is confined to the colon. The CD is responsible for transmural inflammation involving all mucosal layers of the intestinal walls, often resulting in full-thickness inflammation with knife-like fissures. While CD most commonly affects the terminal ileum and the cecum, any part of the GI tract may be involved [52]. Gross pathology of affected areas may exhibit cobblestone mucosa, creeping fat, and strictures. Creeping fat is associated with hyperplastic growth of adipose tissue and mesenteric fat accumulation, correlating with the severity of transmural inflammation [53].

The typical age of onset occurs in a bimodal distribution, with a peak at 15-35 years and another at 55-70 years of age. It is associated with a higher prevalence in individuals of Northern European descent and those of Ashkenazi Jewish descent [54].

Clinical features of CD include weight loss, diarrhea, and abdominal pain. Patients typically present with chronic diarrhea and abdominal pain of the right lower quadrant (terminal ileum most commonly affected). The CD is also associated with several extraintestinal manifestations, including the skin, joints, and eyes [55]. This may lead to enteropathic arthritis, uveitis, and erythema nodosum or pyoderma gangrenosum.

Complications include malabsorption, such as failure to thrive, anemia, and weight loss. Additionally, perianal and enterocutaneous fistulas may develop and are typically associated with abscess formation. Noncaseating granulomas are seen in CD, a collection of macrophages resulting from inflammation without necrosis.

UC

UC is characterized by chronic mucosal inflammation of the rectum including colon and cecum. Affected areas demonstrate mucosal and submucosal ulcers. UC begins in the rectum and can extend to the cecum continuously while the remainder of the GI tract remains unaffected. Inflammation causes crypt abscesses with neutrophils. The gross appearance of affected areas reveals pseudopolyps and loss of haustra [56].

There is a higher prevalence in white populations and those of Ashkenazi Jewish descent, with a peak incidence of 15-35 years of age [57]. There is a genetic predisposition with HLA-B27, a specific allele of class 1 major histocompatibility complex associated with seronegative arthropathies such as ankylosing spondylitis [58].

Dysregulation of the immune system is a critical component in the development of UC. Upregulation of lymphatic cell activity, including plasma cells, B cells, and T cells in the bowel, leads to the formation of an immune reaction. This leads to a cytotoxic effect on the colonic epithelium, resulting in inflammation and local tissue damage, such as erosions, necrosis, and ulcerations within the mucosa and submucosa. Chronic inflammation begins in the rectum and spreads continuously proximally throughout the colon up to the cecum. This inflammation, however, is limited to the mucosa and submucosa [59].

Pathophysiology of IBD

The pathogenesis of IBD significantly involves the dysregulation of the intestinal immune system and its response to microbiota. The intestinal epithelium is a barricade that prevents bacteria or antigens from entering the circulation. In IBD, this barrier becomes defective due to primary barrier function failure or severe inflammation. Protective mechanisms are compromised, including mucus production by goblet cells and secretion of antimicrobial α-defensins by Paneth cells. Excessive inflammatory reactions lead to ongoing epithelial damage, increasing exposure to intestinal microbes and further exacerbating inflammation [60]. Studies have shown that excessive IL-17 production plays a crucial role in IBD progression. Th17 cells secrete IL-17, which is central to this process [61]. Inhibiting Th17 cells can reduce acute colitis by lessening inflammation [62]. Lymphocytes in IBD are responsible for actively initiating and maintaining inflammation, which results in gut tissue damage. Colonic lesions in IBD patients exhibit significant immune cell infiltration and tissue devastation [60].

Numerous cytokines and chemokines are linked to IBD development. For example, the IL-1 family is critical in IBD pathogenesis. IL-1β promotes inflammation in UC, originating from monocytes and macrophages and expressed in the colonic mucosa [60]. IL-18 is elevated in CD patients' mucosa and enhances the Th1 response. IL-33 stimulates mucus secretion to protect the epithelium and increases IL-5 and IL-13 expression as part of the Th2 response. IL-6, which activates STAT3, plays a significant role in inflammation and is elevated in UC and CD patients. TNF-α is vital in IBD pathogenesis, correlating with disease severity [60]. IL-10, an immunosuppressive cytokine, has therapeutic potential despite inconsistent levels in IBD patients. TGF-β has dual roles in IBD, promoting epithelial repair and fibrosis while inducing tolerance and homeostasis through immunoregulation [60].

IL-17, a pro-inflammatory cytokine that activates STAT3, drives chronic inflammation in IBD. IL-17 mRNA levels are increased in the inflamed mucosa of both UC and CD patients. IL-17 has several isoforms, including IL-17A and IL-17F [60]. While inhibiting IL-17A can reduce inflammation, its role in IBD is debated. IL-17A inhibition decreases acute colitis inflammation but can reinforce tight junctions and protect epithelial cells [60].

Nonetheless, IL-17 is a significant inflammatory factor in CD pathogenesis, with higher levels in CD patients. IL-17 increases T cell recruitment into the lamina propria during inflammation. Chemokines like IL-8, which attract neutrophils, are also elevated in IBD patients, contributing to inflammation [60].

**Figure 3:**
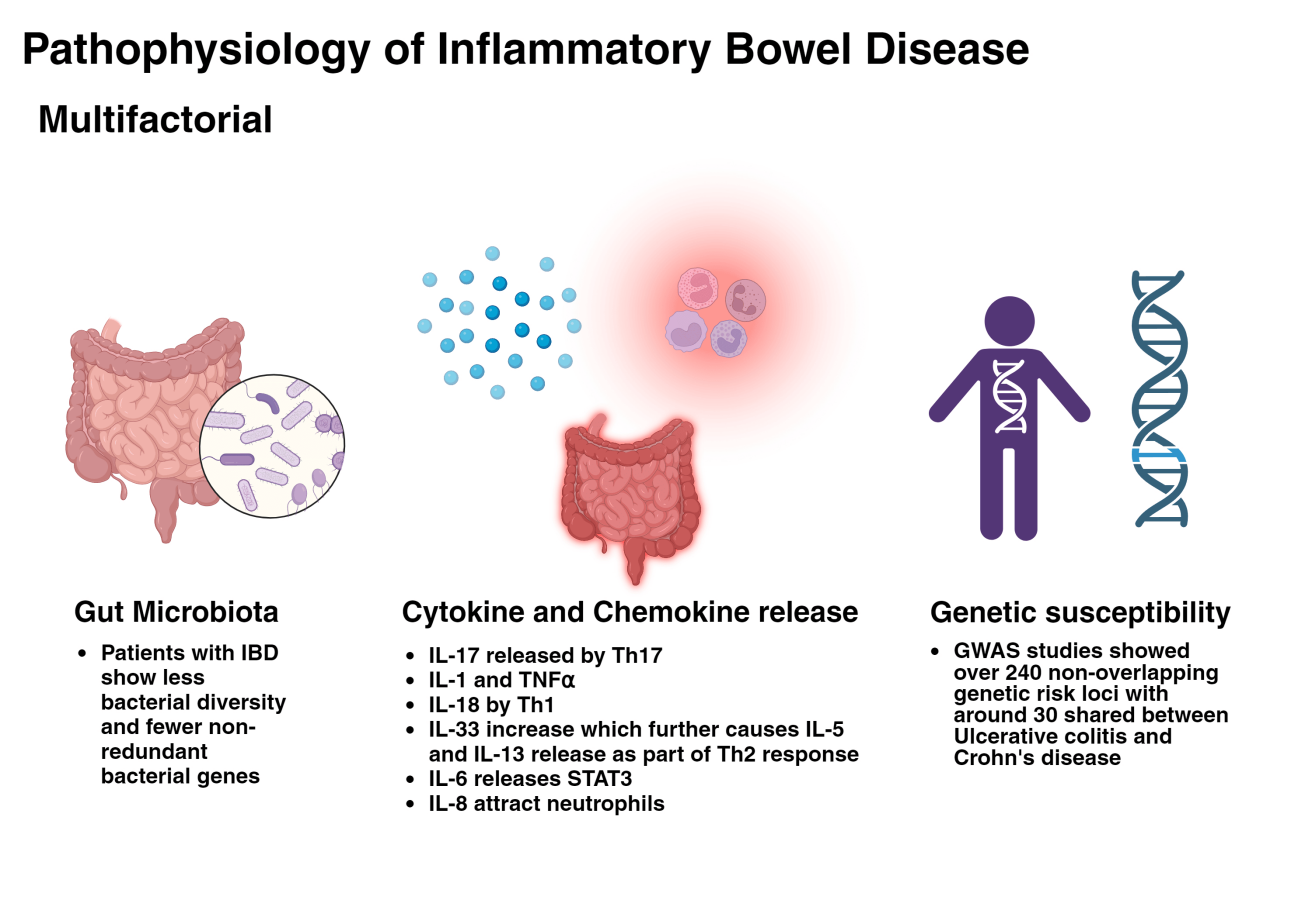
Pathophysiology of inflammatory bowel disease Reference: [60] GWAS: genome-wide association studies; IBD: inflammatory bowel disease

Th17 cells are recognized as critical pathogenic factors in IBD, with increased Th17 cells and IL-17-related cytokines in the inflamed tissues of IBD patients. Various cytokines, such as IL-1β, IL-6, IL-21, IL-23, and TGF-β, influence Th17 cell differentiation in the intestine [60]. STAT3, a Th17-specific transcription factor, is critical for Th17 differentiation. Th17 cell differentiation and IBD pathogenesis, with specific microbiota, causes Th17 differentiation. Inflammation in IBD involves plastic Th17 cells releasing IL-17, with elevated IL-17 levels in IBD patients' mucosa and serum. GWAS links Th17 genes to UC and CD susceptibility, implicating Th17 signaling in IBD pathogenesis. IL-17A and IL-17F have contrasting roles in colitis models, with IL-17A suppression causing excessive inflammation and IL-17F having protective roles. Current IBD therapies focus on blocking IL-17A and IL-17F [60]. Figure 3 demonstrates the factors causing IBD.

Genetic factors in IBD

Genetic susceptibility to IBD has been identified through GWAS, revealing over 240 non-overlapping genetic risk loci, with around 30 shared between CD and UC [63]. Key CD loci include FOXO3, IGFBP1, and XACT [64-66].

NOD2 mutations are the most potent genetic risk factor in IBD; NOD2 mutations (R702W and G908R) increase susceptibility by impairing immune responses and autophagy in intestinal cells [67-69]. As a member of the Nod-like receptor family, NOD2 detects microbial invaders by recognizing muramyl dipeptide found in bacterial cell walls. This recognition triggers a conformational change in NOD2, activating NF-κB and releasing proinflammatory cytokines like IL-12 and TNF-α [70]. NOD2 also facilitates autophagy, the body's cellular recycling system, which degrades dysfunctional cellular components and pathogens, maintaining intestinal homeostasis and regulating the interaction between innate and adaptive immunity [71].

IBD has also been shown to derive from a deficiency of FOXP3. This gene controls the development of regulatory T cells within the intestines and is responsible for the secretion of inhibitory cytokines such as IL-10, IL-35, and TGF-β. Individuals deficient in FOXP3 have been shown to develop enteropathy, damage, and irritation to the small intestine. Studies also suggest that early onset of IBD may be due to single gene mutations, such as mutations in the IL-10 receptor, both α and β chains [72].

Microbiome in IBD

Similar to psoriasis, IBD patients also show significant dysbiosis, with changes in the microbiome contributing to the disease, particularly in genetically predisposed individuals. This results in the proliferation of facultative anaerobes and invasive *Escherichia coli* strains, particularly in the colon, terminal ileum, and rectum [43]. The microbiota composition in individuals with IBD differs from that in healthy individuals [73-77]. There is decreased biodiversity, reduced Firmicutes, Bacteroidetes, Lactobacillus, and Eubacterium, and fewer butyrate-producing species in IBD patients. Moreover, there is an increase in Enterobacteriaceae, *E. coli*, and Fusobacterium [44]. Butyrate-producing bacteria are crucial for maintaining intestinal homeostasis, as they support the growth of epithelial cells and the differentiation of regulatory T cells [20]. In patients with IBD, there is a noted decrease in the abundance of these bacteria, including *Faecalibacterium prausnitzii *[77]. This decline is vital because butyrate, a key SCFA produced by these bacteria, is essential for intestinal health and inflammation reduction [20-22].

Conversely, there is a relative increase in Proteobacteria, predominantly *E. coli*, in patients with CD, especially in mucosa-associated microbiota compared to fecal samples [75]. A specific type of *E. coli* associated with CD, known as adhesion-invasive *E. coli*, was initially isolated from adult CD patients [78]. The rise in pathogenic bacteria like adherent-invasive *E. coli *(AIEC), which can adhere to the intestinal epithelium, impacts intestinal permeability, alters gut microbiota diversity and composition, and triggers inflammatory responses by regulating the expression of inflammatory genes, ultimately leading to intestinal inflammation [20-22].

The host responds to dysbiosis in IBD by releasing AMPs, reactive oxygen species, and immune mediators and inducing mucosal changes, all aiming to rebalance the microbiota and shape the gut's local ecosystems [79]. Metabolites and species with anti-inflammatory properties that are reduced in IBD are believed to offer protection. In contrast, those with proinflammatory properties and increased presence are linked to causing the disease [80]. Although the microbial component of IBD is widely acknowledged, the exact mechanisms connecting genetics, environment, and gut microbes remain poorly understood [81].

Shared pathophysiological mechanisms

Both IBD and psoriasis present with a variety of symptoms affecting the gut and the skin, respectively, but seem to have similar underlying drivers of multifocal inflammation and disease manifestation. Researchers have proposed a gut-skin-joint axis that suggests how the gut microbiota, disturbed immune balance, and bowel permeability may affect skin and joints, affecting their homeostasis and causing inflammation [5]. A meta-analysis determined that the prevalence of psoriasis in individuals with CD and UC was 3.6% and 2.8%, respectively. Conversely, the prevalence of CD and UC among patients with psoriasis was 0.7% and 0.5%. CD and UC were significantly associated with psoriasis and vice versa [82].

Both autoimmune conditions have a broad spectrum of disease variation and usually peak onset around 15-30 years [83]. Various immunological mechanisms have been recognized in both conditions: impaired barrier function and heightened epithelial cell turnover leading to a leaky barrier; predominant proinflammatory IFN-γ-producing NK cells in disease lesions; the migration of T-reg cells from the bloodstream into inflamed tissues increasing disease risk; and Th-17 lymphocytes are significant for PS and IBD pathogenesis [4].

*Th17 Cells* 

The IL-23/Th17 axis is crucial for the development of both conditions. Research indicates that Th17 cells infiltrate the intestinal mucosa of IBD patients more than in healthy controls, and the levels of the cytokine IL-17, which is secreted explicitly by Th17 cells, are elevated in these patients [66]. Similarly, Th17 cells are found in high levels in psoriasis patients, and their presence is positively associated with the severity of the condition [84].

IL-17 antagonism is a highly effective approach for treating psoriasis. Drugs targeting IL-17, such as IL-17A inhibitors, have shown significant efficacy in reducing psoriatic symptoms [85]. In contrast, the inhibition of IL-17 in IBD has not been as successful. Clinical trials with IL-17 inhibitors in CD have shown no benefit and, in some cases, even led to exacerbation of the disease. This suggests that while IL-17 plays a proinflammatory role in psoriasis, it may be protective in maintaining gut integrity in IBD, making its inhibition less effective or potentially harmful in IBD patients [83,85].

T-reg Cells

T-reg cells, or T-suppressor cells, are a subset of T-cells that specialize in suppressing abnormal immune system activation in various organs, including the intestine and skin. In psoriasis, T-regs have a reduced ability to suppress immune responses, resulting in an imbalanced ratio of T-regs to Th17 cells. This dysfunction of T-regs is linked to the worsening of psoriatic disease [25]. T-regs are significantly impacted in IBD. In IBD patients, the number of T-regs is often reduced in the peripheral blood and inflamed intestinal mucosa compared to healthy individuals [86,87]. Additionally, the function of these Tregs is frequently impaired, contributing to the uncontrolled inflammation characteristic of the disease [87]. Similarly, psoriasis patients show functional and numerical abnormalities of T-regs in peripheral blood and superficial dermis [86].

Genetics

GWAS have demonstrated a link between PS and IBD, identifying 13 loci associated with psoriasis (PSORS1-13) and 28 loci associated with IBD (IBD1-28). The strongest correlations are found at the chromosomal loci 6p22, 16q, 1p31, and 5q33 [4,18,88]. A single nucleotide polymorphism (SNP: rs6908425) in locus 6p22 was linked to CD [89]. Subsequently, an association between locus 6p22 and psoriasis was identified compared to healthy controls [88,90].

The IL-23R gene plays a crucial role in IBD (IBD17) and psoriasis (PSORS7) pathogenesis. This significant gene is situated at locus 1p31.1 [88]. The link between psoriasis (PSORS11) and IBD (IBD19) with locus 5q33.1 is well documented, particularly regarding the polymorphisms of the IL12B gene (also known as IL-23B or p40) [88]. The risk alleles rs6556416 and rs6887695, identified in IBD, have also been found in psoriasis [91,92].

Diagnosis and clinical management

Psoriasis Diagnosis

The diagnosis of psoriasis is mainly clinical, based on the characteristic presentation of the skin lesions. Typically, well-demarcated erythematous plaques with silvery scales on the surface are seen, often associated with pruritus. The plaques in individuals with intensely pigmented skin are usually gray and may result in marked post-inflammatory hyperpigmentation. Although it can affect any body part, lesions frequently appear on elbows, knees, scalp, intergluteal lines, nerves, and back [93].

Occasionally, psoriasis may present with atypical manifestations that mimic other dermatoses, such as discoid lupus erythematosus, eczema, dermatophyte infections, or mycosis fungoides. In these cases of unusual presentation, it is crucial to look for characteristic signs of the disease in commonly affected areas, such as the scalp, behind the ears, around the umbilicus, in the sacral region, or the intergluteal groove. Nail involvement is diagnostic, presenting as pitting, oil stains, onycholysis, or subungual hyperkeratosis. Additionally, two pathognomonic signs can aid in the diagnosis: Koebner's phenomenon, which describes the appearance of psoriatic lesions at sites of cutaneous trauma, and Auspitz's sign, characterized by the appearance of a punctate hemorrhage upon removal of the plaque scales due to trauma to the underlying dermal capillaries [93,94].

The most common form of psoriasis, present in 90% of cases, is psoriasis vulgaris. One-third of these patients experience extensive body surface involvement, with moderate to severe disease. In addition to psoriasis vulgaris, there are four additional variants of the disease: guttate, inverse, pustular, and erythrodermic. The pustular and erythrodermic variants are potentially more severe and aggressive. In pustular psoriasis, pustules form around the plaques, usually on the palms and soles, or all over the body. This form may be accompanied by fever, leukocytosis, and hepatotoxicity. The erythrodermic variant is characterized by generalized edema and redness of the skin, affecting approximately 90% of the body. These patients are susceptible to infections, severe dehydration, and electrolyte imbalances, requiring immediate treatment in a hospital setting [93,94].

In cases where diagnostic doubt persists, a skin biopsy should be done to confirm the diagnosis. Characteristic histopathological findings include acanthosis (thickening of the stratum spinosum), parakeratosis (presence of nuclei in the stratum corneum cells), and thinning of the stratum granulosum. In the stratum corneum, all the neutrophils accumulate, forming Munro's microabscesses [93,94].

Treatment of Psoriasis

Psoriasis is a chronic relapsing disease that persists over time with periods of flare-ups and remissions. Because of this pattern, patients often require long-term therapy. The intensity of psoriasis can be assessed by various scales, the most commonly used being the Psoriasis Area and Severity Index (PASI). This scale classifies psoriasis from mild to severe, depending on the severity of the lesions and the percentage of the body affected. Additionally, PASI allows an objective evaluation of the effectiveness of different antipsychotic drugs [6,9].

Most patients (80%) present with mild to moderate psoriasis, which can be managed with topical therapy. In contrast, moderate to severe psoriasis often requires a combined approach of topical and systemic therapy due to the greater extent of the affected surface area [9].

There are multiple systemic treatment options. Among the oldest are acitretin, cyclosporine, fumaric acid esters, and methotrexate. In the last decade, technological advances have led to the development of small molecules (such as apremilast and tofacitinib) and various biologics, including anti-TNF-alpha, anti-IL-17, anti-IL-23, and anti-IL-12/23 [6,9].

Comorbidities, such as psoriatic arthritis and other inflammatory diseases, are crucial in treatment selection. These associated conditions may influence the choice of drugs that offer simultaneous benefits for multiple conditions, allowing for a more comprehensive and efficient therapeutic approach [6,9].

Microbiota Treatment Approaches in Psoriasis

Emerging therapies such as probiotics, prebiotics, and fecal microbiota transplantation (FMT) have shown promise in modulating microbiota composition and influencing disease outcomes.

In a 12-week open-label, single-center clinical trial, probiotic and prebiotic supplementation improved quality of life and reduced inflammation in psoriasis patients receiving anti-psoriatic treatment. It also led to favorable changes in metabolic markers and enhanced gut microbiota diversity [95]. Another study found that probiotic supplementation significantly improved the quality of life and reduced psoriasis severity in patients, along with notable reductions in pro-inflammatory markers and cardiovascular risk factors, suggesting potential benefits in managing psoriasis and associated health issues [96]. Moreover, a trial with 90 psoriasis patients showed that 66.7% of those in the probiotic group, compared to 41.9% in the placebo group, had a 75% reduction in PASI (p=0.0317). The probiotic group also had fewer relapses (20% vs. 41.9%, p=0.027) and beneficial changes in gut bacteria [97]. Additionally, ingesting prebiotic dietary seaweed extract-sulfated xylorhamnoglucuronan (SXRG84) shows potential for improving certain inflammatory skin conditions by reducing pro-inflammatory cytokines [98,99].

FMT has emerged as a novel therapeutic approach in managing psoriatic arthritis, aiming to modulate the gut microbiome and its systemic effects on immune-mediated inflammation. FMT involves the transfer of healthy donor fecal microbiota to recipients [100], potentially restoring microbial balance and alleviating inflammatory responses implicated in psoriatic arthritis pathogenesis. A study highlighted significant differences in plasma protein signatures between psoriatic arthritis patients and healthy controls, demonstrating FMT's ability to modulate inflammatory markers like IFN-γ [101]. In contrast, a randomized controlled trial showed that while FMT was safe, it did not outperform placebo treatment in reducing treatment failure or improving functional outcomes measured by The Health Assessment Questionnaire Disability Index (HAQ-DI) [102]. This discrepancy underscores the complexity and variability in FMT's efficacy across psoriatic arthritis patients. Additionally, a qualitative study provided valuable patient perspectives, showing that despite clinical outcomes, participants perceived FMT positively, citing an improved understanding of their condition and overall quality of life [103]. These insights highlight the potential for FMT to influence patient experiences beyond clinical metrics, necessitating further research to refine patient selection criteria and treatment protocols in microbiota-targeted therapies for psoriatic arthritis. Table 2 summarizes therapeutic approaches in psoriasis microbiome.

**Table 2:** Therapeutic approaches in psoriasis microbiome: study outcomes FMT: fecal microbiota transplantation; HAQ-DI: Health Assessment Questionnaire Disability Index; HDL: high-density lipoprotein; PASI: Psoriasis Area and Severity Index; PGA: physician global assessment

Study type	Treatment approach	Patient group (sample size)	Main findings	References
Open-label, single-center clinical trial	Probiotics:* Bacillus indicus *(HU36), *Bacillus subtilis* (HU58), *Bacillus coagulans* (SC208), *Bacillus licheniformis* (SL307), and *Bacillus clausii* (SC109). Prebiotics: fructooligosaccharides, xylooligosaccharides, and galactooligosaccharides	Patients with psoriasis receiving topical therapy (63)	Spore-based probiotics and precision prebiotics improved quality of life, reduced inflammation, and restored gut microbiota in psoriasis patients on anti-psoriatic therapy	[95]
Randomized, double-blind, placebo- controlled clinical trial single center	A multi-strain probiotic capsule including* Lactobacillus acidophilus*, *Bifidobacterium bifidum*, *Bifidobacterium lactis*, and *Bifidobacterium langum* with colony forming units	Patients with recent psoriasis diagnosis (50)	Probiotics improve patients' quality of life and inflammatory biomarkers in psoriatic patients	[96]
Randomized, double-blind, placebo-controlled clinical trial	A probiotic mixture of *Bifidobacterium longum* (CECT 7347), *B. lactis* (CECT 8145), and *Lactobacillus rhamnosus* (CECT 8361) with a total of colony-forming units per capsule, formulated on maltodextrin	Patients with plaque psoriasis diagnosis at least one year prior to the study (90)	The probiotic improved the Psoriasis Area and Severity Index and Physician Global Assessment index. Additionally, reduced the relapse risk	[97]
Randomized, double-blind placebo crossover trial	Prebiotic seaweed extract - sulfated xylorhamnoglucuronan (SXRG84)	Patients with inflammatory skin conditions including psoriasis, eczema, or rosacea (44)	Ingesting SXRG84 for six weeks reduced inflammatory cytokines, with some participants experiencing skin improvements	[98]
Double-blind, randomized placebo-controlled trial	Ulva sp. 84-derived sulfated polysaccharide - “xylorhamnoglucuronan” (SXRG84)	Overweight or obese participants (64)	Significant reduction in non-HDL cholesterol, atherogenic index, and reduction in C-reactive protein; beneficial shifts in gut flora	[99]
Randomized double-blind placebo-controlled crossover trial	Ulva sp. 84-derived sulfated polysaccharide - “xylorhamnoglucuronan” (SXRG84)	Overweight or obese participants (64)	No significant differences in lipid measures; significant reductions in inflammatory cytokine after SXRG84 treatment	
Double-blind, randomized, sham-controlled trial	Gastroscopic-guided healthy donor fecal microbiota transplantation	Patients with moderate-to-high peripheral psoriatic arthritis disease activity, despite at least 3 months of methotrexate treatment (31)	Patients with active psoriatic arthritis have a distinct immunological plasma protein signature compared with healthy control pre-fecal microbiota transplantation. FMT affects several of these disease markers, including sustained elevation of IFN-γ	[101]
Double-blind, parallel-group, placebo-controlled, superiority trial	Fecal gastroscopic-guided microbiota transplantation into the duodenum	Adults with active peripheral psoriatic arthritis despite ongoing treatment with methotrexate (31)	FMT did not result in serious adverse events but showed higher treatment failure rates compared to sham. Improvement in HAQ-DI favored the sham group	[102]
Qualitative study nested within a double-blind, randomized, placebo-controlled trial	Fecal microbiota transplantation	Patients with psoriatic arthritis (10)	Participation in the study improved patients' understanding of psoriatic arthritis and positively impacted their daily lives. They felt renewed hope and enhanced care	[103]

Inflammatory bowel disease diagnosis

IBD presents a variable clinical spectrum, with symptoms that range from mild to severe during relapses and that may remit partially or entirely during periods of remission. While UC is characterized by chronic inflammation confined to the mucosa of the colon and rectum, CD is more widespread and affects any segment of the gastrointestinal tract [104].

The diagnosis of IBD is based on a comprehensive evaluation comprising multiple essential components. It begins with an assessment of clinical findings, followed by a thorough biochemical evaluation, including complete blood count, inflammatory markers (CRP and ESR), serum electrolytes, and liver enzymes. Stool analysis is crucial to rule out common pathogens and detect Clostridium difficile toxin. Endoscopic studies, such as sigmoidoscopy and colonoscopy, are essential to examine the mucosa and obtain biopsies of detected lesions. In addition, further imaging studies such as ultrasound, computed tomography, and abdominal magnetic resonance imaging are required to determine the extent and severity of the disease, as well as to detect complications [105,106].

On the other hand, markers can be of great help. One of the most sensitive indicators of intestinal inflammation in IBD patients is fecal calprotectin, a neutrophil-derived protein. Its levels correlate with endoscopic indices to gauge disease activity, making it valuable for initial diagnosis, detection of relapses, and assessment of response to treatment. However, it lacks specificity for discriminating between IBD and other causes of intestinal inflammation. Other neutrophil-derived proteins, such as elastase, lysozyme, and lactoferrin, may also be useful as markers of inflammation [105,106].

Research suggests that the debut of IBD at a young age (<40 years) is associated with a worse prognosis in both CD and UC. In CD, this early onset leads to an increased risk of progression to disabling disease, characterized by complications such as strictures, fistulas, and abscesses. This translates into higher rates of surgery, hospitalization, corticosteroid dependence, and disease recurrence. Early diagnosis in the case of UC correlated with a more aggressive clinical course, manifested by a higher frequency of relapses, an increased need for colectomy, and an elevated risk of developing colorectal cancer in the long term [107]. These findings underline the crucial importance of an early and accurate diagnosis followed by an individualized therapeutic approach. The goal is to modify the natural history of the disease, preventing intestinal damage, reducing long-term complications, and positively impacting patients with IBD [107].

Treatment of inflammatory bowel disease

The treatment of IBD is to achieve and maintain remission, prevent new flare-ups, and modify the natural course of the disease. The therapy is developed according to the severity, extent, and behavior of the disease, as well as the individual patient's response [108].

UC

The traditional therapeutic approach in UC follows a stepwise approach, adapted to the severity of the disease. Aminosalicylates constitute the first line of treatment, especially effective in mild to moderate cases of UC. Corticosteroids are introduced when there is no adequate response to aminosalicylates or in moderate to severe cases. They are effective in inducing remission but are not recommended as maintenance therapy due to their long-term adverse effects. The dose should be gradually reduced once clinical response is achieved. Immunomodulators are used in patients who do not respond to corticosteroids, who require frequent courses of them, or who cannot be tapered off them because of the recurrence of symptoms. They include thiopurines (azathioprine, 6-mercaptopurine) and methotrexate.

Biological Therapies

They include anti-tumor necrosis factor (TNF) (infliximab, adalimumab, golimumab), anti-integrins (vedolizumab), and anti-IL12/23 (ustekinumab). They are used in moderate to severe disease or, in some cases, refractory to conventional therapies. JAK inhibitors, such as tofacitinib, are approved for moderate to severe UC.

This stepwise approach is complemented by adjuvant therapies such as antidiarrheals, analgesics, nutritional support, and probiotics according to the patient's needs. The choice and sequence of medications should be personalized, considering the disease severity and previous treatment response [107,108].

CD

Pharmacologic management for CD is divided into two main phases: the induction phase and the maintenance phase.

In the induction phase, acute flares are managed using agents with rapid onset of action, such as corticosteroids and biological therapies. These treatments should be continued until there is objective evidence of remission. Clinical evidence of improvement usually occurs within two to four weeks and, at most, within 12 to 16 weeks. Remission may be clinical, biochemical, histological, and endoscopic. However, asymptomatic patients may have persistent inflammatory changes on endoscopy. Endoscopic evidence is currently the best indicator of remission of IBD. In addition, non-invasive markers of intestinal inflammation, such as fecal calprotectin and cross-sectional imaging, can be used as suitable alternatives to assess remission [107,108].

The maintenance phase is used to maintain disease remission. Immunomodulators and biological therapies are the main agents of maintenance therapy, which is usually continued for a prolonged period [107,108]. Table 3 summarizes medication for CD.

**Table 3:** Medications for the management of Crohn's disease CD: Crohn's disease

Disease severity	Medications		Reference
	Induction of remission	Maintenance	
Mild to moderate	Oral corticosteroids. Consider enteral nutritional therapy. Consider the use of sulfasalazine if there are lesions in the colon. TNF inhibitors are recommended to be considered for steroid-dependent or refractory patients.	Asymptomatic patients and/or those with a low risk of CD progression: Provide supportive therapy as needed. Patients at high risk of CD progression or with ongoing inflammation: Consider using anti-TNF-alpha therapy.	[106,107]
Moderate to severe	Oral corticosteroids. Consider enteral nutritional therapy. TNF inhibitors are recommended to be considered for steroid-dependent or refractory patients. If pharmacotherapy or nutrition therapy is ineffective or unable to adapt, the combination with granulocyte monocyte apheresis (GMA) can be considered.	Gradually reduce and then discontinue corticosteroids. Maintain the use of non-steroidal agents that achieved remission. Combination therapy with anti-TNF-α antibodies and an immunomodulator is preferred over using monotherapy.	[106,107]
Severe to fulminant	It is recommended that patients be hospitalized, given infusions and blood transfusions if needed, and administered antibiotics if infection is suspected. Systemic Corticosteroids (if no active infection). TNF inhibitors are recommended to be considered for steroid-dependent or refractory patients. Seek early surgical consultation for patients in poor general condition who do not respond to anti-TNF agents.		

Recently, in the management of IBD, a "top-down" or accelerated treatment approach has been adopted in patients with poor prognostic factors or severe disease at debut. This approach consists of early initiation of more potent therapies, such as immunomodulators or biologics, preventing complications and reducing the need for surgery [107,108].

The treatment choice in IBD should be individualized, considering factors such as the severity and extent of the disease, the presence of extraintestinal manifestations, and associated comorbidities. Medical management is the cornerstone of treatment; however, surgery becomes a necessary therapeutic option in certain circumstances. Surgery is considered when, despite optimal medical management, adequate disease control is not achieved or when the patient's life is affected by disease progression, complications, or adverse effects of drug treatment. In addition, surgical intervention is indicated urgently in critical situations such as colonic perforation, massive bleeding, toxic megacolon, or when diagnosed with colorectal cancer or high-grade dysplasia [107,108].

Once disease control is achieved, close follow-up is essential. This should include periodic clinical assessments, inflammatory biomarker monitoring, and endoscopic evaluations to ensure sustained remission, early detection of relapses, and timely treatment adjustment. This comprehensive and personalized approach optimizes therapeutic outcomes and improves patients' long-term quality of life with IBD [107,108].

Microbiota treatment approaches in IBD

Probiotics

In a double-blinded, placebo-controlled pilot study, the efficacy of probiotics on oxidative stress in IBD patients was evaluated. The probiotic group showed significant improvements in oxidative stress markers and overall antioxidant response compared to the placebo group. This suggests that specific probiotics are effective and safe for managing oxidative stress in IBD patients [109]. A double-blind, placebo-controlled pilot study assessed the effects of microencapsulated sodium butyrate on the gut microbiota of IBD patients. Results showed that sodium butyrate supplementation increased the growth of beneficial bacteria producing SCFA, potentially exerting anti-inflammatory effects [110].

Dietary Interventions

A cross-over study investigated the impact of a low-fat, high-fiber diet (LFD) versus an improved standard American diet (iSAD) on UC patients' quality of life, inflammation markers, and intestinal dysbiosis. Participants' quality of life improved with both diets, but the LFD also significantly reduced markers of inflammation and dysbiosis. The study concluded that dietary interventions, particularly an LFD, can benefit UC patients in remission by reducing inflammation and improving gut microbiota composition [111]. Additionally, a study investigated the efficacy of three variants of the specific carbohydrate diet (SCD) in managing active CD. Eighteen pediatric patients with mild to moderate CD were enrolled and randomized into SCD, modified SCD (MSCD), or a whole foods (WF) diet group. Over the 12-week study period, all participants who completed the study achieved clinical remission, emphasizing the therapeutic potential of dietary interventions. Reductions in C-reactive protein levels across all groups indicated decreased inflammation, with more restrictive diets (SCD and MSCD) showing greater improvements than the WF diet. Additionally, significant shifts in microbiome composition were observed among all patients, underscoring the diet's impact on gut microbial communities [112].

FMT

FMT is a promising therapeutic approach in IBD. In a pilot randomized controlled study on CD patients, FMT did not meet its primary endpoint of donor microbiota implantation but showed promising results in maintaining clinical remission compared to sham transplantation. Specifically, FMT significantly decreased the CD Endoscopic Index of Severity at six weeks and prevented increased C-reactive protein levels, suggesting a potential role in reducing inflammation [113]. Conversely, in a placebo-controlled trial focused on active UC, FMT demonstrated efficacy in inducing remission, with a 17% higher remission rate than placebo [114]. These findings support further exploration of FMT as a therapeutic option for managing IBD, emphasizing its potential in modulating the gut microbiome to achieve clinical improvements.

Therapeutic synergies and overlaps

IBD and psoriasis share common inflammatory pathways, which makes it possible to consider simultaneous therapeutic strategies for both conditions [115,116].

TNF-a and IL-12/23 inhibitors have been shown to treat both diseases effectively. However, paradoxical psoriasis reactions have been documented in IBD patients managed with TNF-a inhibitors, especially in the pediatric population. Also, among TNF-a inhibitors, etanercept is the only one not approved for IBD due to studies suggesting a two-fold increased risk of developing UC and CD [115,116].

On the other hand, IL-17 inhibitors have been successfully used for the management of psoriasis and psoriatic arthritis. However, their use has been linked to an increased rate of IBD onset or exacerbation, according to multiple studies [116-118].

Numerous non-biologic systemic medications used to treat psoriasis, including tacrolimus, leflunomide, cyclosporine, methotrexate, sulfasalazine, azathioprine, 6-thioguanine, and mycophenolate mofetil, have been shown to have potential for treating IBD. Additional clinical research is necessary to ensure their safety and effectiveness [115,116].

New molecules such as risankizumab and recombinant IL-25 and IL-37 proteins are currently under investigation and show promising preliminary results. However, further studies are needed to ascertain their efficacy and safety in treating these conditions [116]. Table 4 summarizes FDA-approved biologic agents for IBD and psoriasis.

**Table 4:** FDA-approved biologic medications for IBD and psoriasis IBD: inflammatory bowel disease; FDA: Food and Drug Administration; CD: Crohn's disease; UC: ulcerative colitis

Type of drug		Disease				Additional information
		Psoriasis	Psoriatic arthritis	IBD		
				CD	UC	
TNF-a inhibitors	Infliximab	FDA approved [119]	FDA approved [120]	FDA approved [121]	FDA approved [122]	Paradoxical psoriasis (PXPS) reactions have been reported after treatment, especially in the pediatric population [116,123,124].
	Adalimumab	FDA approved [125]	FDA approved [126]	FDA approved [127]	FDA approved [128]	
	Golimumab	Phase III clinical trial for psoriatic arthritis suggests efficacy in psoriasis [129]	FDA approved [129]	Small open-label study suggests efficacy [130]	FDA approved [131]	
	Certolizumab pegol	Phase II clinical trial suggests efficacy [132]	FDA approved [133]	FDA approved [134]	Small open-label study suggests limited efficacy [135]	
	Etanercept	FDA approved [136]	FDA approved [137]	Tested, not approved for IBD due to studies suggesting an increased risk of developing UC and/or CD [115,116,138,139]		Not effective in IBD [140]
IL-12/23 inhibitors	Ustekinumab	FDA approved [141]	FDA approved [142]	FDA approved [143]	No clinical trials available	A few paradoxical psoriasis reactions and arthritis flares, after Ustekinumab have been described. As well as efficient Ustekinumab treatment in paradoxical psoriasis reactions [115,116,123]
IL-23 inhibitors	Guselkumab	FDA approved [144]	Phase II and two phase III trials, have proven safety and efficacy for psoriatic arthritis as well [145-147]	The results from ongoing studies have not been published yet.		A few case reports describe optimistic results in CD [148-150]
IL-17 inhibitors	Secukinumab	FDA approved [151]	FDA approved [152]	Phase II clinical trial suggest s lack of efficacy. Potential risk of exacerbation of IBD [153,154]		Recent evidence suggests that IL-17 inhibitors may induce or exacerbate already existing IBD [115,116,153-156].
	Ixekizumab	FDA approved [155]	Phase III clinical trial suggests efficacy [156]	Potential risk of exacerbation [155]		
	Brodalumab	FDA approved [157,158]	Phase II data suggests efficacy [159,160]	Contraindicated based on worsening symptoms [161]	No clinical trials available	

Therapeutic implications and challenges

The involvement of IL-23 in the pathophysiological pathways of IBD and psoriasis has been established. Anti-IL-23 medications like certolizumab, adalimumab, and infliximab have made remission possible for this disease. Conversely, anti-IL-17 medications show promise in treating UC but are unsuccessful in treating CD [4]. Research on anti-IL-35 drugs is necessary since the application of these drugs on mice has shown an effective remission rate. Further evidence showing that the development of psoriatic lesions is linked to anti-TNF-α therapy highlights the need to comprehend the precise disease process rather than just the drug sensitization [115].

It has been historically challenging to establish the precise reason why psoriasis patients are more likely to develop CD than UC. Similarly, a connection is seen with diseases, including rheumatoid arthritis, non-melanocytic skin cancers, and mental health disorders [162]. Investigating the intestinal microbiome in inflammatory bowel illnesses is crucial, particularly about *F. Prausnitzii*, which has been the subject of fecal microbiota transplants in psoriasis patients who have not responded to methotrexate-based treatment [163].

Understanding the genetic relationships between psoriasis and CD, such as those found at 9p24 near JAK2, 10q22 in ZMIZ1, 11q13 near PRDX5, 16p13 near SOCS1, 17q21 in STAT3, 19p13 near FUT2, and 22q11 in YDJC, is essential for the development of future genetic relocation therapies [1,164]. In the future, tailored treatment through precision medicine with particular biomarkers would control the underlying pathology while expecting a decrease in side effects from patients' response to the drug.

Quality of life and psychological impact

Psoriasis

Psoriasis, a non-communicable, autoimmune, and incurable disease, is often associated with high levels of distress, which are frequently under-recognized. Patients with palmoplantar, pustular, and arthropathic psoriasis generally experience poorer quality of life. Psychological issues, including poor self-esteem, sexual dysfunction, anxiety, depression, and suicidal ideation, are prevalent and can vary based on the affected body regions, such as the scalp, face, neck, forearms, hands, and genital areas. Notably, the clinical severity of psoriasis does not always reflect its emotional impact [165-167].

Public misconceptions about psoriasis can exacerbate feelings of shame and embarrassment, leading to severe mental health issues and increased disability [168,169]. Patients with psoriasis often suffer from poor sleep quality and have a significantly higher risk of insomnia and restless legs syndrome. These conditions are associated with more severe psoriasis, systemic inflammation, and reduced quality of life [170,171]. Additionally, decreased self-esteem due to depression, anxiety, and stress is common among adults with psoriasis. Comorbid depression is linked to drug addiction, alcohol use, and sexual dysfunction, particularly in patients with genital lesions, who report significantly worse quality of life [172,173].

Psoriasis during childhood or adolescence can significantly affect personality development, leading to long-term psychological morbidity [174]. Therefore, there is a greater need for psychological care in young people with psoriasis. Even successful treatment may not alleviate psychological distress, as patients with psoriasis and their coping mechanisms are crucial. Providing psychotherapeutic support to enhance self-acceptance and prevent depression is essential [167,174]. Simple screening tools and psychological interventions have shown promise in recent trials [167].

Addressing the psychosocial needs of psoriasis patients starts with early identification, which can be challenging. Once identified, outcomes can be improved through effective inflammation treatment, cognitive behavioral therapy, meditation, mindfulness-based therapy, and psychotropic medication [175].

IBD

Patients with IBD often experience significant food-related quality of life impairment, regardless of disease type and activity. There is a notable correlation between IBD and eating disorders, including anorexia nervosa and avoidant restrictive food intake disorder (ARFID), as well as orthorexia nervosa [176,177]. Psychosocial factors, including affective status and fear around eating, impact quality of life. Profiling IBD patients based on food index and health engagement can help clinicians identify those at greater risk of maladaptive food-related behaviors [178].

Fatigue is prevalent in IBD patients and is associated with disease activity, sleep quality, depression, and anxiety. Factors such as female gender, obesity, and opioid use increase the risk of active IBD and poor mental health profiles while being over 40 years old is associated with a lower risk [179]. IBD patients often experience disturbed sleep, which may serve as a marker for subclinical disease activity. Studies suggest circadian misalignment in IBD patients, necessitating further research into its clinical implications [180].

Anxiety and depression are common in IBD. Perceived stress is linked to mood disturbances in both UC and CD. Anxiety in UC is associated with perceived stress and a new diagnosis of IBD, while depression is related to stress, inpatient status, and active disease. In CD, anxiety is linked to perceived stress, abdominal pain, and lower socioeconomic status, and depression to perceived stress and increasing age [181].

Underdiagnosed and under-treated mental health disorders in IBD patients can negatively impact outcomes, disease management, and medication adherence and increase healthcare utilization and associated costs [182,183]. Identifying mood disorders and providing stress management and psychotherapy are essential for optimizing disease outcomes and improving quality of life [181,182,184]. Antidepressants have been shown to improve depressive symptoms and quality of life in IBD patients compared to placebo, with SNRIs also potentially improving anxiety [185]. Further studies on the role of antidepressants in IBD are needed, and IBD-specific cognitive behavioral therapy can be successfully implemented [186].

While pediatric IBD can pose psychological challenges, many young patients cope well with their disease, indicating significant individual variation in well-being. This variation underscores the need for comprehensive supportive measures [187,188,189].

## Conclusions

The review concludes the similarities between inflammatory bowel disease (IBD) and psoriasis, accentuating the familiar underlying drivers of multifocal inflammation and disease manifestation with a gut-skin-joint axis proposed to explain their interconnection. Both conditions are associated with TH17 cells and T-reg cells, with IL-17 antagonism effective in psoriasis but not IBD, and alterations in the gut microbiome impacting inflammation and disease activity. As both diseases share common inflammatory pathways, it allows the possibility of simultaneous therapeutic strategies. Non-biologic systemic medications used to treat psoriasis can also treat IBD, but additional clinical research is required to ensure their safety and effectiveness. New molecules such as risankizumab and recombinant IL-25 and IL-37 proteins show promising preliminary results, but further studies are necessary to confirm their efficacy and safety profile.
